# Risk factors of incomplete balloon-assisted enteroscopy with an analysis of 943 patients: a retrospective study

**DOI:** 10.3389/fmed.2025.1612974

**Published:** 2025-07-01

**Authors:** Jun Liu, Meng Wan, Peng Wang, Jing Guo, Yan-Bo Yu, Xiu-Li Zuo

**Affiliations:** ^1^Department of Gastroenterology, Shandong University Qilu Hospital, Jinan, Shandong, China; ^2^Shandong Provincial Clinical Research Center for Digestive Diseases, Jinan, Shandong, China

**Keywords:** complete enteroscopy, risk factors, balloon-assisted enteroscopy, double-balloon enteroscopy, single-balloon enteroscopy

## Abstract

**Background:**

Balloon-assisted enteroscopy (BAE) plays an important role in the diagnosis and therapy of small bowel diseases. Complete enteroscopy is considered an objective quality indicator of enteroscopy. However, there are limited studies on the factors associated with complete BAE. This study aimed to determine the factors affecting complete BAE.

**Methods:**

All adult patients with indications for BAE were investigated at a tertiary medical center from January 2019 to December 2022. Their medical records and BAE procedure-associated data were reviewed and analyzed. Risk factors of incomplete enteroscopy were investigated using univariate analysis and multivariable logistic regression analysis.

**Results:**

A total of 943 patients meeting the eligibility criteria were analyzed. Among these, 558 patients achieved complete enteroscopy. In multivariable logistic regression analysis, single-balloon enteroscopy (SBE) [odds ratio (OR) = 2.35, 95% confidence interval (CI): 1.79–3.09, *p* < 0.001], male sex (OR = 1.62, 95% CI: 1.22–2.15, *p* = 0.001), intestinal surgery (OR = 2.26, 95% CI: 1.79–3.09, *p* = 0.003), and body mass index (BMI) ≥ 28 kg/m^2^ (OR = 1.20, 95% CI: 1.07–1.34, *p* = 0.002) were independent predictors of incomplete enteroscopy.

**Conclusion:**

This retrospective study identified SBE, male sex, intestinal surgery, and BMI ≥ 28 kg/m^2^ as independent risk factors for incomplete enteroscopy.

## 1 Introduction

Balloon-assisted enteroscopy (BAE) is an effective endoscopic technique used for diagnosing and treating small bowel diseases. Two types of BAE, double-balloon enteroscopy (DBE) and single-balloon enteroscopy (SBE), are commercially available. BAE is not available in many endoscopy units, as it is time-consuming and requires specialized training.

The length of the adult small bowel averages around 600 cm (1), and the diagnostic and therapeutic yields of BAE depend on the depth of small bowel insertion and complete vision of the entire gastrointestinal tract for many patients (2–4). Complete enteroscopy is considered an objective quality indicator of enteroscopy and the most comprehensive method for the visualization of the small bowel (5). A higher rate of complete enteroscopy is associated with a greater chance of detecting meaningful gastrointestinal lesions. Complete enteroscopy ensures confirmation of the presence, variety, number, and distribution of lesions throughout the small intestine, which have a positive influence on further management and surveillance of the small bowel (3, 6–8). Patients with obscure gastrointestinal bleeding (OGIB) who achieved complete endoscopy had a lower chance of rebleeding than those without complete examination (9). However, limited data exists on the factors associated with the completion or insertion depth of BAE.

This study aimed to investigate the risk factors associated with incomplete enteroscopy. Identifying these factors may help to achieve hierarchical patient management and endoscopic procedure planning.

## 2 Materials and methods

### 2.1 Patients

This retrospective study was conducted at a tertiary medical center (Qilu Hospital of Shandong University, Jinan, China). Consecutive adult patients who underwent BAE from January 2019 to December 2022 were reviewed. Patients who met any of the following criteria were excluded: (1) cases terminated upon the target lesion (strictures, masses, hemorrhagic or other lesions) where further insertion was clinically unnecessary; (2) need for therapeutic intervention, including hemostasis, polypectomy, removal of foreign body in the small bowel, lithotripsy, enteroscopy-assisted endoscopic retrograde cholangiopancreatography, and enteroscopy-assisted jejunostomy tube placement, making further insertion unnecessary; (3) risk of bleeding due to fragile gastrointestinal mucosa, gastrointestinal varices, and Mallory–Weiss syndrome; (4) poor bowel preparation; (5) patients with an insertion route of intestinal stoma; and (6) incomplete clinical data and endoscopic information.

The decision for complete enteroscopy as an examination endpoint was made by the endoscopist after understanding the patient’s clinical data, the purpose of the enteroscopy, and endoscopic findings. This study was approved by the Medical Ethics Committee of Qilu Hospital of Shandong University (No. KYLL-202401-030-1).

### 2.2 BAE

BAE was performed with DBE (EN-580 T enteroscopy, Fujifilm, Japan) or SBE (SIF-Q260 enteroscopy, Olympus Optical, Japan), both using a sliding overtube (ST-SB1, Olympus, Tokyo, Japan) and PB-10 pressure controlled pump system (Olympus, Tokyo, Japan). All patients were instructed to fast for 12 h before the procedures, and bowel preparation (polyethylene glycol electrolyte solution mixed with 2 L water) was administered 4–5 h before enteroscopy. General anesthesia (intravenous propofol, 2–3 mg/kg/h) was administered by anesthesiologists monitoring cardiorespiratory parameters. All patients had intubation and mechanical ventilation. CO_2_ insufflation was used during all enteroscopic procedures. X-ray fluoroscopy guidance was used in some patients who had difficulty with endoscope insertion. To explore the influence of enteroscopy operators’ experience, we tried our best to collect the learning curves of each operator for DBE and SBE.

All patients were scheduled for both antegrade and retrograde procedures. The initial insertion path of the enteroscopy was determined based on clinical information, previous findings, or preference of the endoscopist. If scope advancement was unsuccessful despite applying abdominal pressure or changing the patient’s position, the procedure was terminated. Endoscopic tattooing was performed at the furthest insertion point as a landmark. The opposite route was subsequently carried out. Complete enteroscopy was confirmed if the cecum was reached via the oral route, the duodenal papilla was reached via the anal route, or the tattoo mark made by the initial procedure was reached via the opposite route.

### 2.3 Data collection

We retrospectively reviewed and analyzed the following variables: patients’ sex, age, height and body mass index (BMI); history of abdominal and pelvic surgery; history of diabetes, smoking and alcohol consumption; indications for enteroscopy (OGIB, small bowel stricture or obstruction, diagnosed or suspected polyposis/tumor, diagnosed or suspected Crohn’s disease, abdominal pain, diarrhea, and other reasons); types of enteroscopy (DBE or SBE); and the initial and subsequent insertion routes.

### 2.4 Statistical analysis

Continuous variables were expressed as mean ± standard deviation (SD) and compared using Student’s *t*-test. Categorical variables were presented as frequencies and percentages and compared using the chi-square test or Fisher’s exact test. Factors with *p* < 0.10 in univariate analysis were included in the multivariable logistic regression analysis. Odds ratios (ORs) with 95% confidence intervals (CIs) were reported. *p* < 0.05 was considered statistically significant. Statistical analyses were performed using SPSS 26.0 (IBM Corp., Armonk, NY, United States).

## 3 Results

### 3.1 Patient characteristics

**Figure 1:**
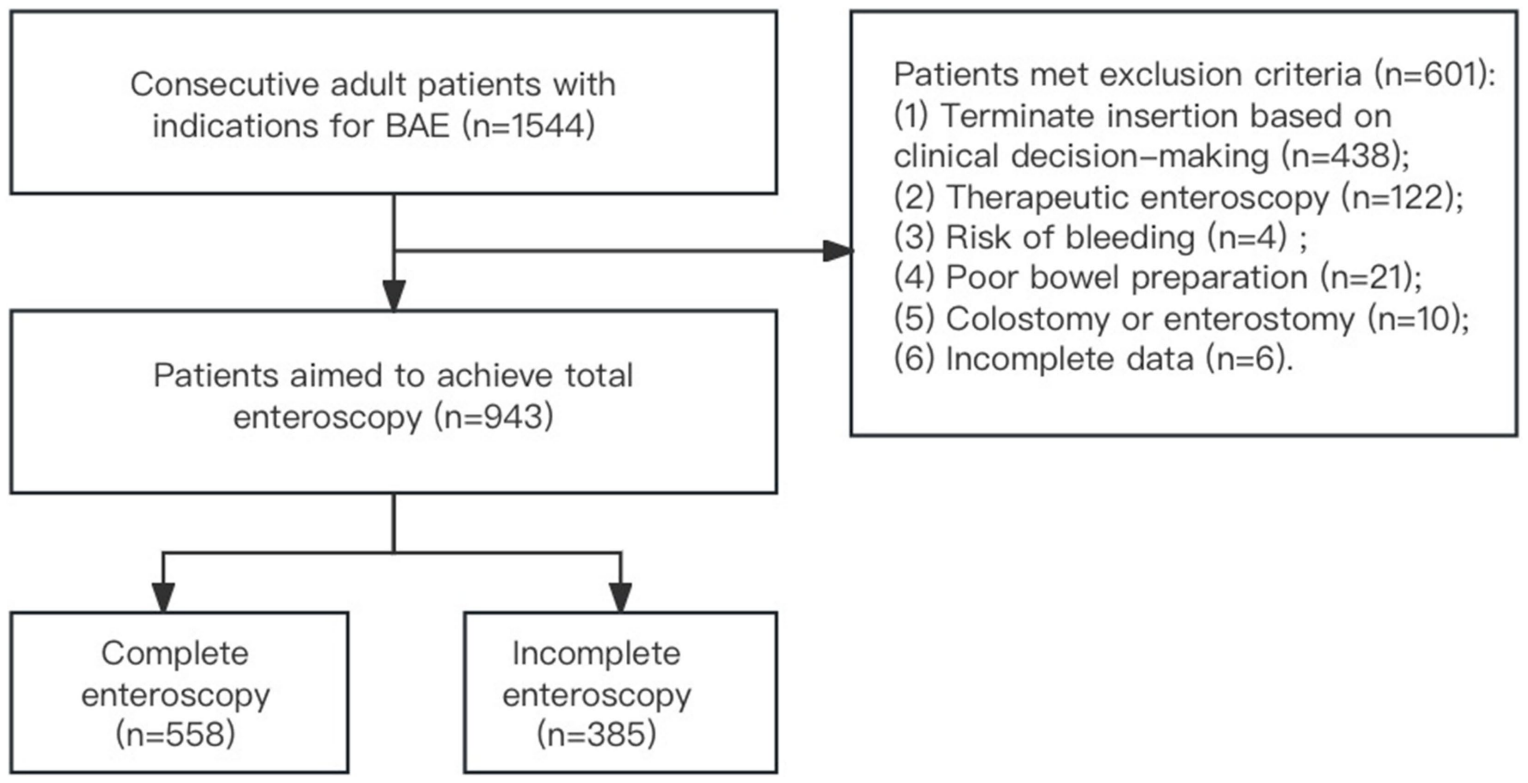
Flowchart of patient recruitment.

From January 2019 to December 2022, BAE was performed on 1,544 patients with suspected small bowel diseases at Qilu Hospital of Shandong University. Among these patients, 438 were excluded for terminating further insertion based on clinical decision-making, 122 for therapeutic intervention, 21 for poor bowel preparation, 10 for previous colostomy or enterostomy surgery, six for incomplete clinical data and endoscopic information, and four for risk of bleeding. A total of 943 patients were ultimately included in the analysis. Among these, 558 patients achieved complete enteroscopy, while 385 did not (Figure 1).

The baseline characteristics of the patients are summarized in Table 1. The mean age was 49.78 ± 15.0 years, and 60.9% were male. Indications for enteroscopy were OGIB (32.3%, 305/943), small bowel stricture or obstruction (16.0%, 151/943), diagnosed or suspected polyposis/tumor (6.6%, 62/943), diagnosed or suspected Crohn’s disease (24.1%, 227/943), abdominal pain (12.6%, 119/943), diarrhea (3.9%, 37/943), and other reasons (4.5%, 42/943). About 8.2% (77/943) of patients had diabetes.

**Table 1:** Baseline characteristics of patients with suspected small bowel diseases aiming at complete enteroscopy by BAE.

Characteristics of patients (*n* = 943)	No.
Sex (male/female)	574/369
Age (yrs.), mean ± SD	49.78 ± 15.00
Indication	
OGIB	305 (32.3%)
Small bowel stricture or obstruction	151 (16.0%)
Polyposis or tumor	62 (6.6%)
Diagnosed or suspected Crohn’s disease	227 (24.1%)
Abdominal pain	119 (12.6%)
Diarrhea	37 (3.9%)
Other reasons	42 (4.5%)
Diabetes	77 (8.2%)
Gastrectomy	13 (1.4%)
Intestinal surgery	61 (6.5%)
Appendectomy	56 (5.9%)
Cholecystectomy	17 (1.8%)
Hernia surgery	20 (2.1%)
Hysterectomy	22 (2.3%)
Smoking	261 (27.7%)
History of alcohol consumption	312 (33.1%)
Height (m), mean ± SD	1.67 ± 0.08
Types of enteroscopy	
DBE	491 (52.1%)
SBE	452 (47.9%)
Initial insertion routes	
Oral route	917 (97.2%)
Anal route	26 (2.8%)
Learning curve	
≤50 produces	184 (19.5%)
>50 produces	759 (80.5%)

SD, standard deviation; OGIB, obscure gastrointestinal bleeding; DBE, double balloon enteroscopy; SBE, single balloon enteroscopy.

The rates of different abdominal and pelvic surgical history were as follows: gastrectomy (1.4%, 13/943), intestinal surgery (61/943, 6.5%), appendectomy (5.9%, 56/943), cholecystectomy (1.8%, 17/943), hernia surgery (2.1%, 20/943), and hysterectomy (2.3%, 22/943). The rate of smoking was 27.7% (261/943), and 33.1% (312/943) had a history of alcohol consumption. SBE accounted for 47.9% (452/943), while DBE accounted for 52.1% (491/943). The oral route was used as the initial insertion route in 97.2% (917/943), and the anal route in 2.8% (26/943). A total of 184 (19.5%) patients were from the operators’ first 50 cases, and 759 (80.5%) patients were from the operators’ subsequent cases.

### 3.2 Univariate analysis

Among the 943 patients, 558 (59.2%) achieved complete enteroscopy. Potential predictive factors were assessed by univariate analysis (Table 2). Eight of them had *p*-values less than 0.10, including SBE (*p* < 0.001), male sex (*p* < 0.001), small bowel stricture or obstruction (*p* = 0.026), intestinal surgery (*p* = 0.003), smoking (*p* = 0.002), alcohol consumption (*p* = 0.013), greater height (*p* = 0.002), and BMI ≥ 28 kg/m^2^ (*p* = 0.003). The learning curve of operators (≤50 vs. >50 cases) did not significantly affect the completion rates of enteroscopy (*p* = 0.250) (Table 2).

**Table 2:** Univariate analysis for risk factor of incomplete enteroscopy.

Variables	Complete enteroscopy (*n* = 558)	Incomplete enteroscopy (*n* = 385)	*P*
Types of enteroscopy, *n* (%)			<0.001
SBE	221 (39.6%)	231 (60.0%)	
DBE	337 (60.4%)	154 (40.0%)	
Initial insertion routes			0.335
Oral route	545 (97.7%)	372 (96.6%)	
Anal route	13 (2.3%)	13 (3.4%)	
Learning curve			0.250
≤50 produces	102 (18.3%)	82 (21.3%)	
>50 produces	456 (81.7%)	303 (78.7%)	
Sex			<0.001
Male	309 (55.4%)	265 (68.8%)	
Female	249 (44.6%)	120 (31.2%)	
Age	50.20 ± 14.97	49.14 ± 15.04	0.286
Indication			0.167
OGIB	191 (34.2%)	114 (29.6%)	0.136
Small bowel stricture or obstruction	77 (13. 8%)	74 (19.2%)	0.026
Polyposis/tumor	37 (6.6%)	25 (6.5%)	0.933
Diagnosed or suspected Crohn’s disease	132 (23.7%)	95 (24.7%)	0.719
Abdominal pain	77 (13.8%)	42 (10.9%)	0.189
Diarrhea	18 (3.2%)	19 (4.9%)	0.184
Other reasons	26 (4.7%)	16 (4.2%)	0.712
Diabetes	42 (7.5%)	35 (9.1%)	0.389
Gastrectomy	7 (1.3%)	6 (1.6%)	0.694
Intestinal surgery	25 (4.5%)	36 (9.4%)	0.003
Appendectomy	34 (6.1%)	22 (5.7%)	0.809
Cholecystectomy	8 (1.4%)	9 (2.3%)	0.305
Hernia surgery	9 (1.6%)	11 (2.9%)	0.192
Hysterectomy	13 (2.3%)	9 (2.3%)	0.994
Smoking	134 (24.0%)	127 (33.0%)	0.002
History of alcohol consumption	167 (29.9%)	145 (37.7%)	0.013
Height (m), mean ± SD	1.67 ± 0.08	1.68 ± 0.08	0.009
BMI ≥ 28 kg/m^2^	41 (7.3%)	51 (13.2%)	0.003

SBE, single balloon enteroscopy; DBE, double balloon enteroscopy; OGIB, obscure gastrointestinal bleeding; SD, standard deviation; BMI body mass index.

### 3.3 Logistic multivariable analysis

Logistic multivariable regression analysis identified the following as independent predictors of incomplete enteroscopy: SBE (OR = 2.35, 95% CI: 1.79–3.09, *p* < 0.001), male sex (OR = 1.62, 95% CI: 1.22–2.15, *p* = 0.001), intestinal surgery (OR = 2.26, 95% CI: 1.79–3.09, *p* = 0.003), and BMI ≥ 28 kg/m^2^ (OR = 1.20, 95% CI: 1.07–1.34, *p* = 0.002) (Table 3).

**Table 3:** Logistic multivariate analysis for risk factor of incomplete enteroscopy.

Variables	OR (95%CI)	*P*
Types of enteroscopy (SBE)	2.35 (1.79–3.09)	<0.001
Male	1.62 (1.22–2.15)	0.001
Small bowel stricture or obstruction	–	0.059
Intestinal surgery	2.26 (1.31–3.90)	0.003
Smoking	–	0.331
History of alcohol consumption	–	0.642
Height	–	0.479
BMI ≥ 28 kg/m^2^	1.20 (1.07–1.34)	0.002

SBE, single balloon enteroscopy; BMI, body mass index.

## 4 Discussion

The introduction of BAE has improved the diagnosis and treatment of small bowel diseases. More lesions are being detected as the depth of insertion into the small bowel has increased (3). However, achieving complete enteroscopy is challenging due to the deep position and tortuous anatomy of the small bowel loops, and incomplete visualization of small bowel mucosa may partially account for missed pathologic lesions during BAE (10, 11). Prospective studies have reported a high heterogeneity in the percentage of complete enteroscopy during BAE, ranging from 0 to 92% (12–17). Thus, identifying the risk factors of incomplete enteroscopy is important in evaluating the diagnostic and therapeutic yield, as well as the time and risks associated with individual patients (18).

OGIB is the most common indication for small bowel visualization, and performing complete enteroscopy becomes essential in these cases (2). Similar to previous studies, the present study found that OGIB was the major indication. Uncertainties persist regarding the predictors of complete enteroscopy during BAE. Nevertheless, several factors have been implicated in previous studies, including a history of abdominal surgery, indications for enteroscopy, BMI, CO_2_ insufflation, and types of BAE (SBE or DBE) (19, 20). The present study demonstrated that SBE, male sex, intestinal surgery, and BMI ≥ 28 kg/m^2^ were independent predictors of incomplete BAE. The identification of these risk factors may aid with clinical procedure planning and a definite diagnosis, reduce the burden of the endoscopist, and decrease the procedure time of enteroscopy.

In a randomized controlled trial published in 2011, complete enteroscopy was more easily performed with DBE than with SBE, although the study was limited by a small sample size (13). In a more recent study, the complete enteroscopy rate of SBE was reported to be lower than that for DBE. However, the difference was not significant (21). Similarly, our findings, based on a large sample size (452 with SBE, 491 with DBE), also demonstrated DBE with a higher complete enteroscopy rate.

Sex was previously reported as a significant predictor of complete enteroscopy (22). This is consistent with our findings, indicating that male sex is an independent predictive factor for incomplete enteroscopy. It is well known that there is sexual dimorphism in body fat distribution. Men generally have more abdominal visceral adipose tissue than women (23), which may partially explain this result. Previous literature has shown that women are less likely to achieve complete colonoscopy (24), a finding that contrasts with findings in enteroscopy and should be noted by endoscopists.

A history of previous abdominal surgery can affect the depth of maximal insertion (DMI) during DBE, and there is a strong association between the number of abdominal surgeries and the DMI for both anterograde and retrograde approaches (20). The present study investigated the influence of abdominal and pelvic surgery in detail by categorizing procedures into gastrectomy, intestinal surgery, appendectomy, cholecystectomy, hernia surgery, and hysterectomy. Intestinal surgery was an independent negative factor for incomplete enteroscopy, while other abdominal and pelvic surgeries showed no significant difference. This may be explained by mesenteric adhesions limiting the mobility of the small bowel and creating areas of fixed angulations, which are difficult to negotiate even using flexible BAE.

Obesity has been postulated as a negative predictive factor for DMI by limiting the length of bowel that can be pleated on the overtube of BAE. However, the definite influence of BMI has not been rigorously studied (25). One study indicated a significant linear relationship between small bowel length and height, while the relationship between small bowel length and BMI, although also linear, was not significant (1). In the current study, obesity (BMI ≥ 28 kg/m^2^) was identified as an independent risk factor for incomplete BAE. Greater height was also identified as a risk factor for incomplete enteroscopy; however, it did not remain significant in multivariable logistic analysis.

Prior studies on the DMI or complete DBE did not identify age as an influencing factor, which was consistent with our findings (20, 26). In our study, an indication of small bowel stricture or obstruction was not an independent risk factor for incomplete enteroscopy in multivariable logistic analysis. This may be explained by our exclusion criteria regarding terminating examinations for patients with luminal stricture or huge mass, which may increase the complete enteroscopy rate of patients with small bowel stricture or obstruction indication. Previous studies have rarely reported the influence of smoking and alcohol consumption on complete enteroscopy. To our knowledge, our study is the first to investigate the relationship of these two factors with complete enteroscopy. While univariate analysis showed a trend toward higher incomplete enteroscopy rates in patients with a history of smoking and alcohol consumption, these associations were not statistically significant on multivariable logistic analysis. Most patients with smoking and alcohol consumption were male, which may have been a confounding factor in the univariate analysis.

Previous studies on the learning curve of enteroscopy were inconsistent. Gross et al. showed that the complete enteroscopy rose from 8% in the first 50 DBEs, to 63% in the last 50 of 200 DBEs (18). Whereas Tee et al. found that there was no learning curve for transoral DBE (27). Dutta et al. reported a learning curve for transoral SBE, but not for the transanal approach (28). In our endoscopy center, operators are required to have performed over 10,000 gastroscopies and colonoscopies before being permitted to independently perform enteroscopy procedures. We speculate that this rigorous pre-training may have partially mitigated the conventional learning curve of enteroscopy.

This study has some limitations. First, it was a single-center retrospective study. Second, the oral route was the initial insertion route in 97.2% of cases, which was primarily due to the habits and preferences of the endoscopists at our institution. This may partly explain the difference between the findings of the present study and those of previous studies, which demonstrated a higher complete enteroscopy rate by the retrograde approach (6).

## 5 Conclusion

The present retrospective study identified SBE, male sex, intestinal surgery, and BMI ≥ 28 kg/m^2^ as independent risk factors for incomplete enteroscopy. Identification of these predictive risk factors may contribute to the implementation of additional measures to reduce the rate of incomplete BAE, thereby improving the diagnostic efficiency and therapeutic yield of BAE.

## Data Availability

The raw data supporting the conclusions of this article will be made available by the authors, without undue reservation.
